# 累及中枢神经系统多发性骨髓瘤患者的病例对照研究

**DOI:** 10.3760/cma.j.issn.0253-2727.2022.12.007

**Published:** 2022-12

**Authors:** 璐 李, 中原 冯, 沛 郭, 海燕 何, 静 卢, 进 刘, 琬婷 强, 华 姜, 鹃 杜, 卫军 傅

**Affiliations:** 海军军医大学第二附属医院血液科，全军骨髓瘤与淋巴瘤疾病中心，上海 200003 Department of Hematology, Myeloma & Lymphoma Center, Second Affiliated Hospital of Naval Medical University, Shanghai 200003, China

**Keywords:** 多发性骨髓瘤, 中枢神经系统, 治疗, 预后, Multiple myeloma, Central nervous system, Treatment, Prognosis

## Abstract

**目的:**

探讨累及中枢神经系统多发性骨髓瘤（CNS-MM）患者的临床特征、预后因素及治疗。

**方法:**

回顾性分析2011年1月至2022年1月在海军军医大学第二附属医院血液科确诊的18例CNS-MM患者在初诊及CNS受累时的临床资料，并与同时期按1∶3配对的初诊MM患者（对照组）比较，分析两组患者的临床特征、生存差异。

**结果:**

CNS-MM自初诊时的中位发病时间为14.2（0.9～79.6）个月，中位总生存（OS）时间为30.5个月，累及CNS后的中位OS时间仅3.8个月。与对照组相比，CNS-MM患者IgD型多见（*P*＝0.010）、贫血更严重（*P*＝0.014）、骨髓浆细胞比例更高（*P*＝0.013）、髓外病灶多见（*P*＝0.001）、乳酸脱氢酶（LDH）增高（*P*＝0.009）。基于来那度胺或泊马度胺联合方案的血液学+CNS缓解率优于硼替佐米或达雷妥尤单抗联合方案（75.0％对16.7％，*P*＝0.019）。同时存在2个高危因素（高LDH、髓外病灶）的初诊患者，接受含免疫调节剂的方案可显著降低CNS-MM发生率（*P*＝0.026）。初诊时高LDH（*P*＝0.008，*HR*＝7.319，95％*CI* 1.663～32.219）、存在髓外病灶（*P*＝0.006，*HR*＝8.054，95％*CI* 1.828～35.486）是发生CNS-MM的独立危险因素。

**结论:**

CNS-MM患者预后不佳。初诊伴有高LDH或髓外病灶的患者发生CNS-MM的概率增高。基于免疫调节剂的联合治疗可能改善CNS-MM患者的预后。

## Abstract (en)

**Objective:**

To investigate the treatment options for multiple myeloma patients with central nervous system involvement（CNS-MM）, as well as their clinical characteristics and prognostic factors.

**Methods:**

Between January 2011 and January 2022 our center diagnosed 18 people with CNS-MM. A retrospective analysis was done on the clinical information from the initial diagnosis and central nervous system involvement, and it was compared to 1∶3 matched newly diagnosed MM from the same period. Analysis was done on the clinical characteristics and survival rates of the two groups.

**Results:**

In patients with CNS-MM, the median time of onset was 14.2（0.9–79.6）months and the median overall survival（OS）was 30.5 months from initial diagnosis and only 3.8 months in patients after CNS involvement. The CNS-MM patients showed more IgD type（*P*＝0.010）, severer anemia（*P*＝0.014）, a higher proportion of bone marrow plasma cells（*P*＝0.013）, more extramedullary lesions（*P*＝0.001）, and increased lactic dehydrogenase（LDH）（*P*＝0.009）when compared to the control group. Lenalidomide or pomalidomide-based combinations had higher rates of hematology and CNS remission than bortezomib or daratumumab-based regimens（75.0％ *vs* 16.7％, *P*＝0.019）. Patients who received IMiD-based regimens and had 2 high-risk factors at initial diagnosis（high LDH and extramedullary lesions）had a significantly lower incidence of CNS-MM（*P*＝0.026）. At the initial diagnosis, LDH（*P*＝0.008, *HR*＝7.319, 95％*CI* 1.663–32.219）and extramedullary lesions（*P*＝0.006, *HR*＝8.054, 95％*CI* 1.828-35.486）were independent risk factors for the occurrence of CNS-MM.

**Conclusion:**

Patients with CNS-MM had a poor prognosis. Patients with high LDH or extramedullary lesions at the time of the initial diagnosis are more likely to have CNS-MM. The prognosis of this patient may be improved by immunoregulator-based therapy.

多发性骨髓瘤（MM）累及中枢神经系统（CNS）是一种较为罕见的髓外浸润类型，多见于复发进展患者，仅占MM的1％[1]–[2]。文献报道该类患者的预后差[3]–[4]，治疗手段相对匮乏，且目前对其临床特征、疾病危险因素等尚不明确。因此，我们对本中心确诊的18例累及CNS的MM患者以及同期初诊MM患者的临床资料进行配对分析，以探讨该特殊类型患者的临床特征、预后因素及有效治疗手段。

## 病例与方法

1. 病例：纳入2011年1月至2022年1月在海军军医大学第二附属医院血液科确诊的18例累及CNS的MM患者，回顾性分析从初诊到CNS受累时的临床特征。通过随机数表法选取初诊时间相近的未累及CNS的MM患者（±1.5个月）为对照组，按1∶3配对进行病例对照研究。收集患者诊断时、CNS受累时的相关实验室数据、体征、治疗线数、细胞遗传学资料。

2. 诊断：CNS-MM确诊标准：符合国际骨髓瘤工作组（IMWG）MM诊断标准；脑脊液（CSF）形态学检出异常浆细胞或CSF流式细胞术（FCM）检测到单克隆浆细胞，或颅内组织活检证实为浆细胞瘤。

CNS-MM临床诊断标准：符合IMWG MM复发进展标准；CSF形态学或FCM检测未见单克隆浆细胞，但出现CNS受累的临床表现及影像学依据；肿瘤抗原筛查及CT、PET-CT排除CNS外的其他肿瘤。

3. 治疗：12例患者接受基于泊马度胺或来那度胺联合地塞米松的治疗方案，包括P-CBD（泊马度胺、环磷酰胺、硼替佐米、地塞米松）+依托泊苷（Vp16）+甲氨蝶呤（MTX）、P-Cd（泊马度胺、环磷酰胺、地塞米松）、RCD（来那度胺、环磷酰胺、地塞米松）+Vp16+MTX、P-DECP（泊马度胺、顺铂、依托泊苷、环磷酰胺、地塞米松）、R-CBD（来那度胺、环磷酰胺、硼替佐米、地塞米松）、K-Pd（卡非佐米、泊马度胺、地塞米松）、R-V-DECP（来那度胺、硼替佐米、顺铂、依托泊苷、环磷酰胺、地塞米松）、Dara-Pd（达雷妥尤单抗、泊马度胺、地塞米松）；6例接受以硼替佐米或达雷妥尤单抗联合地塞米松为基础的治疗方案，包括V-DECP（硼替佐米、顺铂、依托泊苷、环磷酰胺、地塞米松）、VD（硼替佐米、地塞米松）、V-DT-PACE（硼替佐米、顺铂、依托泊苷、环磷酰胺、地塞米松、多柔比星、沙利度胺）、Dara-DECP（达雷妥尤单抗、顺铂、依托泊苷、环磷酰胺、地塞米松）、Dara-VD、Dara-VD +CTX+Vp16。其中2例患者疗效达非常好的部分缓解（VGPR）后接受了自体外周血造血干细胞移植（ASCT）。所有18例患者在明确诊断CNS-MM后，接受MTX 10 mg+地塞米松5 mg 2次/周或阿糖胞苷100 mg+地塞米松5 mg 2次/周鞘内注射治疗，疾病控制后予以每月1次鞘内注射维持治疗。

4. 疗效评估及生存期：血液学缓解依据IMWG疗效标准评定，分为完全缓解（CR）、部分缓解（PR）、疾病稳定（SD）、疾病进展（PD）。CNS缓解：CSF涂片或FCM未检出浆细胞，同时实质病灶缩小50％（PR）或完全消失（CR）。总生存（OS）时间：自确诊为MM至死亡或末次随访的时间；CNS-MM后的OS时间：自确诊为CNS-MM至死亡或末次随访的时间。

5. 随访：采用住院、门诊病历查阅或电话等方式随访。随访时间截至2022年1月1日。

6. 统计学处理：两组分类变量及数值变量的比较分别使用卡方检验和Mann-Whitney检验；非正态分布数据用中位数（范围）表示；正态分布数据用均值±标准差表示；采用Kaplan-Meier法及Log-rank检验比较两组的OS。采用Cox比例风险模型进行生存的影响因素分析。采用单因素、多元Logistic回归分析发生CNS-MM的危险因素。*P*值<0.05为差异有统计学意义。

## 结果

1. CNS-MM患者临床特征：本组18例CNS-MM患者累及CNS均发生于MM疾病进展期。中位治疗线数为2线，自初诊时的中位发病时间为14.2（0.9～79.6）个月，中位CSF蛋白量595（270～16 200）mg/L。

CNS受累时1例患者表现为癫痫小发作；7例患者出现眼睑下垂、口角歪斜、复视、听力受限、眼球活动外展受限；2例患者出现大小便失禁或尿潴留；8例患者出现肢体运动功能受限，步态不稳表现。

CNS累及部位详见表1。18例患者中8例接受了CSF FCM检测，其中6例CD56表达缺失。依据CSF细胞学结果，分为CSF（+）：脑脊膜型，指CSF涂片见异常浆细胞或FCM检出单克隆浆细胞；CSF（−）：脑实质型，指影像学发现脑实质浸润，但CSF涂片未见异常浆细胞或FCM未检出单克隆浆细胞。

**表1:** 18例累及中枢神经系统多发性骨髓瘤（CNS-MM）患者的临床特征

临床特征	CSF（+）（12例）	CSF（−）（6例）
年龄[岁，*M*(范围)]	56（48~70）	48（39~71）
初诊至CNS累及的时间[月，*M*(范围)]	13.7（0.9~79.6）	14.5（3.5~36.1）
中位治疗线数	2	2
前期治疗方案[例(%）]		
PI	8（66.7）	4（66.7）
PI+IMiD	4（33.3）	2（33.3）
脑脊液蛋白[mg/L，*M*（范围）]	728(270~16 200)	431(303~709)
影像学提示的累及部位[例(%）]		
脑膜/脊膜	6（50.0）	2（33.3）
鞍旁、蝶窦等	2（16.7）	2（33.3）
脑实质	4（33.3）	2（33.3）
CNS外的髓外病灶[例(%）]	6（75.0）	4（66.7）
自初诊时的中位OS时间（月）	28.4	30.5
自CNS-MM诊断时的中位OS时间（月）	7.7	2.9

注 CSF：脑脊液；PI：蛋白酶体抑制剂；IMiD：免疫调节剂；OS：总生存

2. CNS-MM患者初诊与同期初诊MM的临床特征比较：与同期初诊MM患者相比，CNS-MM患者IgD型更多见（27.8％对5.6％，*P*＝0.010），λ轻链型略占优。CNS-MM患者初诊时贫血更严重［HGB（92±32）g/L对（98±23）g/L，*P*＝0.014］、中位骨髓浆细胞比例更高（64％对29％，*P*＝0.013）、髓外侵犯多见（61.1％对18.5％，*P*＝0.001）及中位LDH增高（384 U/L对187 U/L，*P*＝0.009）。两组的中位年龄、M蛋白分类、R-ISS分期、高危细胞遗传学异常、移植的比例及其他实验室检查差异均无统计学意义（*P*值均>0.05）。

3. 疗效、生存与预后：截至末次随访，18例患者中仅3例存活。其中2例均为VGPR时接受ASCT患者，移植后RVD方案维持治疗至今，末次随访仍处于严格意义的CR，且1例达骨髓微小残留病阴性（<10^−6^），2例患者存活时间分别为15.6个月、13.4个月。另1例存活患者为单纯CNS复发者，接受PCd方案治疗后CNS-MM达到CR，但10个疗程后CNS-MM再次复发，经塞替派联合Pd方案治疗1个疗程后再次CR，截至末次随访时OS时间为16.4个月。

自确诊为MM的中位随访时间为27.8（4.2～81.5）个月。与对照组相比，CNS-MM患者OS时间显著缩短（30.5个月对77.5个月，*P*<0.001）（图1），而CNS-MM后的中位OS时间仅3.8个月。

**图1:**
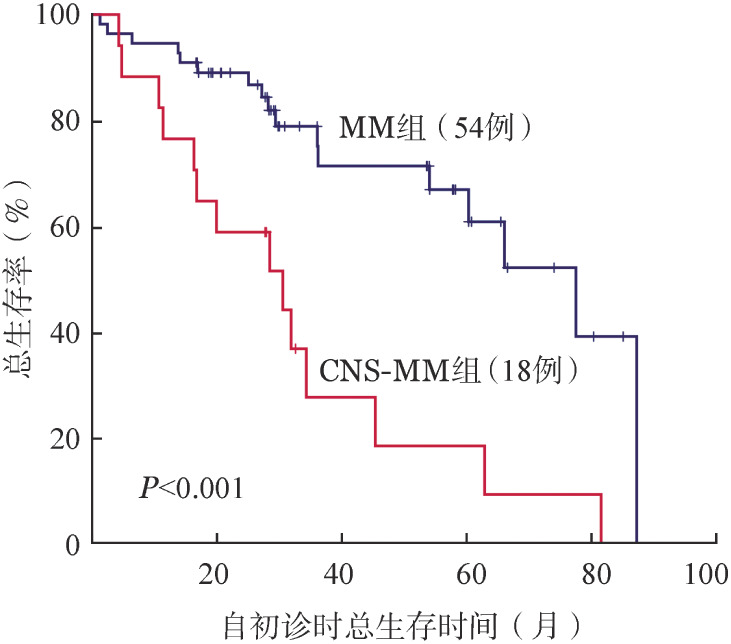
累及中枢神经系统多发性骨髓瘤（CNS-MM）患者与同期初诊MM患者的总生存时间比较

将治疗方案分为2组：方案1基于泊马度胺或来那度胺的联合方案；方案2基于硼替佐米或达雷妥尤单抗的联合方案。两组CNS缓解率分别为66.7％、33.3％（*P*＝0.180），血液学+CNS缓解率分别为75.0％和16.7％（*P*＝0.019）。2线、≥3线患者CNS-MM后的中位OS时间分别为7.7个月和2.9个月（*P*＝0.500）。

Logstic单因素回归分析提示IgD型、初诊时骨髓浆细胞>35％、LDH升高、髓外病灶是发生CNS-MM的危险因素（图2）。多因素分析显示：高LDH（*P*＝0.008，*HR*＝7.319，95％*CI* 1.663～32.219）、初诊时存在髓外病灶（*P*＝0.006，*HR*＝8.054，95％*CI* 1.828～35.486）是发生CNS-MM的独立危险因素。将初诊时血红蛋白、LDH、骨髓浆细胞比例、髓外病灶及疾病过程中是否发生CNS-MM纳入Cox多因素回归分析，结果显示CNS受累是影响患者生存的独立预后因素（*P*＝0.022，*HR*＝3.154，95％*CI* 1.178～8.442）。

**图2:**
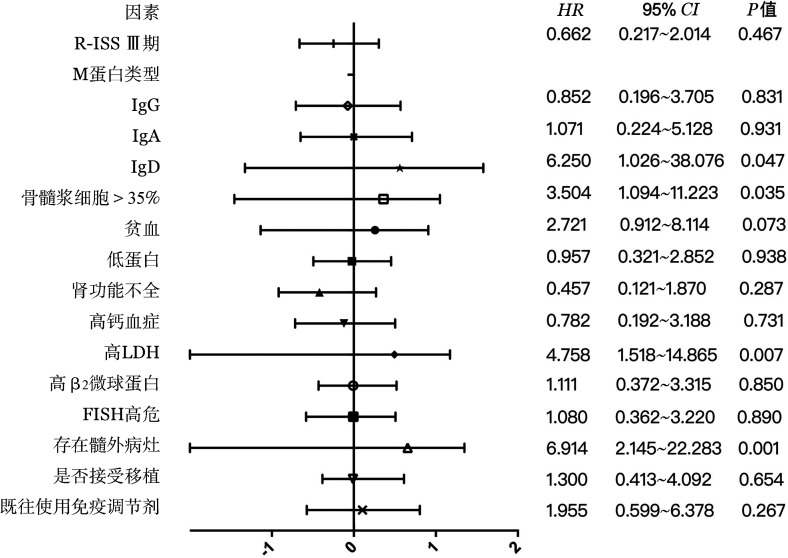
累及中枢神经系统多发性骨髓瘤相关风险因素

初诊时同时具有高LDH及髓外病灶的高危患者（26例），接受含来那度胺或泊马度胺等免疫调节剂（IMiD）方案治疗组的CNS-MM发生率明显低于未接受IMiD治疗组（33.3％对53.3％，*P*＝0.026）。

## 讨论

MM累及CNS主要表现为单个或多个脑实质内病变和（或）轻脑膜累及。临床上将CSF中存在浆细胞和（或）轻脑膜、硬脑膜、脑实质受累，或通过影像学检查评估并经CSF分析、MRI、CT和（或）组织活检证实的患者定义为CNS-MM[5]。鉴于脑、脊髓实质浸润患者的CSF中也可无克隆性浆细胞，故病灶的病理检查仍是诊断的金标准，增强MRI、CT影像学检查和CSF常规是脑、脊髓实质浸润诊断的重要补充[6]–[10]。基于影像学诊断标准，本组患者44.4％有脑膜浸润，55.6％为脑实质受累，未见脑膜、脑实质同时受累者。如以CSF细胞学判断有无脑膜浸润，脑膜型CNS-MM则占66.7％。8例影像学诊断脑膜浸润患者中6例（75％）CSF脱落细胞学或FCM检出异常或单克隆浆细胞。另2例细胞学阴性的患者，可能与早年未采用FCM检测有关。而10例影像学诊断为脑实质浸润患者中，有6例CSF细胞学阳性。上述结果提示，CSF细胞学（FCM）对脑膜浸润的诊断更为敏感，脑膜型CNS-MM的实际占比可能更高。如复发难治MM出现CNS症状、体征、CSF蛋白水平增高，CSF的FCM检测不失为排查MM CNS浸润的有效手段。无论脑膜是否累及，从初诊时或CNS受累后的OS差异无统计学意义。

MM发生CNS浸润的发病机制尚不明确。文献报道CNS-MM的发病可能与MM细胞CD56失表达、13q/17p缺失及MM的高侵袭性有关[11]–[12]。在本组8例接受了CSF FCM检测的患者中，6例CD56（−），提示CD56可能参与了MM的CNS播散。鉴于本组患者发生CNS浸润时接受了中位2线的治疗，自初诊时的中位发病时间仅为14.2个月，提示CNS-MM的发病与既往治疗可能无关，而与MM初诊时的高侵袭性相关。病例配对研究的结果也表明，相较于同期初诊MM患者，与MM侵袭性相关的指标在CNS-MM患者中更为突出：IgD型更多见（*P*＝0.010）、贫血更严重（*P*＝0.014）、骨髓浆细胞比例更高（*P*＝0.013）、髓外侵犯多见（*P*＝0.001）及LDH增高（*P*＝0.009）。尽管CNS-MM骨髓浆细胞比例更高，但血清M蛋白、受累血清游离轻链反而低于对照组，提示发生CNS-MM患者的浆细胞可能更为幼稚。有意思的是，CNS-MM患者无论在初诊还是出现CNS浸润时，其MM细胞FISH检查高危细胞遗传学异常的比例与对照组差异无统计学意义。这有可能与病例数较少有关，也提示CNS-MM的发病与细胞遗传学异常外的其他侵袭性因素关系更大。多因素分析显示初治时高LDH、伴有髓外病灶是CNS-MM的独立危险因素。

由于大部分药物无法透过血脑屏障，CNS-MM患者的治疗手段有限，预后极差。靶向治疗、化疗、鞘内化疗、局部放疗、HSCT的中位OS时间仅2～6个月[3]–[4]。本组CNS-MM自确诊时的中位OS时间明显短于对照组（*P*<0.001），CNS-MM后的中位OS时间仅3.8个月。多因素分析也提示CNS累及是OS的独立危险因素。鉴于CNS-MM患者全身疾病多处于复发进展阶段，在治疗上需兼顾髓内及CNS病灶。IMiD来那度胺能透过血脑屏障，但CSF浓度较低。泊马度胺的CSF浓度可达血药浓度的39％[13]。本研究中，基于来那度胺或泊马度胺的联合治疗，无论CNS缓解率、血液学缓解率或血液学+CNS缓解率均优于硼替佐米或达雷妥尤单抗为基础的联合方案。但两组自CNS累及起的OS时间差异却无统计学意义。18例患者中获得血液学+CNS CR或PR患者的OS时间显著长于未获得PR者（未达到对11.2个月对2.7个月，*P*＝0.006）。表明在CNS-MM的治疗中，深度的血液学和CNS双重缓解是改善患者OS的关键因素。另外，通过合理选择初诊时的方案降低或避免高风险患者发生CNS-MM也是值得临床探索的方向。本组患者中，初诊时具有2个高危因素（高LDH、髓外病灶），但接受IMiD方案治疗患者后期CNS-MM发生率显著降低（33.3％对53.3％，*P*＝0.026）。因此，对于具有高风险发生CNS-MM的患者，在初诊方案选择时，应当优先考虑含血脑屏障通透性药物的治疗方案。另外，预防性鞘内注射在高风险患者中的临床价值也值得探索。

研究具有抗MM活性且能透过血脑屏障的新型疗法，可能是改善CNS-MM预后的新希望。已有成功用塞替派鞘内注射治疗MM伴CNS浸润的报道[14]。新型蛋白酶体抑制剂Marizomib因具有良好的血脑屏障通透性[15]，也可能成为治疗CNS-MM的有效药物。塞利尼索则在CNS受累的非霍奇金淋巴瘤、恶性脑胶质母细胞瘤中表现出良好的疗效和安全性[16]。此外，鉴于苯达莫司汀、靶向BCMA的新型治疗在髓外浆细胞瘤患者中的良好疗效，也具有治疗CNS-MM的潜能。

CNS-MM尽管发病率不高，但现有方案的总体疗效不佳。研究如何减少或减缓CNS-MM的发生及有效的联合治疗方案，值得临床进一步探索。
